# Artificial Intelligence in Cataract Care: A Narrative Review of Evidence Maturity and Clinical Readiness

**DOI:** 10.1155/joph/8100180

**Published:** 2026-11-01

**Authors:** Anđela Jukić, Josip Pavan, Rajka Kasalica Žužul, Biljana Đapić Ivančić, Tomislav Jukić

**Affiliations:** ^1^ Department of Ophthalmology University Hospital Dubrava Avenija Gojka Šuška 6 Zagreb 10000 Croatia kbd.hr; ^2^ Department of Neurology University Hospital Centre Zagreb Ulica Mije Kišpatića 12 Zagreb 10000 Croatia kbc-zagreb.hr; ^3^ Department of Ophthalmology University Hospital Centre Zagreb Ulica Mije Kišpatića 12 Zagreb 10000 Croatia kbc-zagreb.hr; ^4^ School of Medicine University of Zagreb Šalata 2 Zagreb 10000 Croatia unizg.hr

**Keywords:** artificial intelligence, cataract, cataract surgery, clinical validation, deep learning, intraocular lens, machine learning, telemedicine

## Abstract

Artificial intelligence (AI) is being investigated across the cataract care pathway, but the maturity of the evidence varies substantially between technical benchmark studies, externally validated models, prospective clinical evaluations and tools used in routine care. This narrative review critically summarises AI applications in cataract detection, intraocular lens (IOL) power calculation, perioperative risk assessment, intraoperative support and postoperative follow‐up, with particular emphasis on evidence quality, external validity and clinical readiness. A structured search of PubMed/MEDLINE was conducted for English‐language publications from 1 January 2018 through 31 March 2025, with selective retention of earlier seminal studies relevant to cataract risk prediction, force feedback and ophthalmic robotics. Evidence was classified by maturity, from technical proof of concept and retrospective internal validation to external or multicentre validation, prospective clinical evaluation and demonstrated workflow or patient‐level benefit. The strongest evidence for cataract detection comes from large externally validated image‐based models, whereas smartphone systems remain sensitive to image quality, case mix and deployment setting. Data‐driven IOL formulas can improve refractive prediction in selected datasets, but comparisons are affected by constant optimisation, axial‐length distribution, IOL model, measurement device and paired‐eye dependence. Intraoperative AI remains dominated by phase recognition, segmentation and technical performance assessment, with limited evidence of direct clinical benefit. Postoperative conversational AI has reached multicentre prospective clinical evaluation, although safety depends on escalation pathways, human oversight and local workflow design. Clinical translation of AI in cataract care is therefore emerging but uneven: a limited number of applications have reached external or prospective clinical validation, while many remain retrospective or technical proof‐of‐concept systems. Future adoption should be based on external validation, transparent reporting, prospective clinical utility, postdeployment monitoring and clear regulatory and medicolegal accountability rather than benchmark performance alone.

## 1. Introduction

Cataract is a leading cause of visual impairment and blindness globally; in 2020, an estimated 15 million people aged 50 years or older were blind because of cataract [1]. Cataract surgery is among the most frequently performed surgical procedures worldwide [2]. Population ageing and increasing expectations for refractive precision continue to increase pressure on eye‐care services, motivating interest in digital tools that may improve access, efficiency, consistency and perioperative decision‐making.

Artificial intelligence (AI) is an umbrella term that includes machine learning (ML), in which algorithms learn patterns from data, and deep learning (DL), a subset of ML based on multilayer neural networks [3]. These terms are not interchangeable and are used according to the methodology of the cited study throughout this review. In ophthalmology, AI has achieved clinically meaningful results in areas such as diabetic retinopathy screening, but the evidentiary pathway from a high‐performing model to a safe clinical tool is substantially more demanding than technical accuracy alone.

Several previous reviews have summarised AI applications in cataract surgery, including broad narrative overviews and a 2024 systematic review focused specifically on intraoperative video analysis [2–4]. The need for another review therefore lies not in cataloguing additional AI applications, but in distinguishing what has actually progressed towards clinical use from what remains technically promising but insufficiently validated.

Accordingly, this review provides a clinically oriented synthesis across the complete cataract pathway and explicitly classifies the maturity of evidence. We focus on five questions: (1) has the model been validated beyond its development dataset; (2) does it use clinically meaningful performance metrics and reference standards; (3) has it been prospectively evaluated; (4) has it demonstrated workflow or patient‐level benefit; and (5) is there a plausible regulatory and implementation pathway? This clinical‐readiness perspective is intended to complement, rather than duplicate, prior application‐based reviews.

## 2. Methodology

This article is a structured narrative review rather than a systematic review. The literature search was completed on 31 March 2025 using PubMed/MEDLINE. The core search combined cataract‐related terms with AI terminology and was supplemented by domain‐specific searches for IOL calculation, surgical video analysis, perioperative risk prediction, postoperative monitoring and telemedicine. The exact search strings are provided in Appendix S1 (Supporting Information).

English‐language publications from 1 January 2018 through 31 March 2025 were prioritised. Earlier studies were retained selectively when they represented seminal evidence directly relevant to cataract risk prediction, force‐feedback systems, or ophthalmic robotic assistance. Reference lists of key reviews and primary studies were hand‐searched to identify additional relevant publications.

The literature search identified approximately 85 potentially relevant records. After title and abstract screening, 52 full‐text articles were assessed for eligibility. Records were not retained when they addressed noncataract AI applications without direct translational relevance, reported only training‐set performance, duplicated an earlier report without additional validation, or were purely conceptual. Screening was performed by the first author, and uncertain cases were resolved by discussion with the senior authors; duplicate independent screening was not performed and is not claimed.

Studies were eligible if they evaluated an AI/ML/DL method directly related to cataract detection or grading, IOL power prediction, surgical‐risk estimation, intraoperative video understanding or decision support, surgical performance assessment, or postoperative follow‐up; or if they provided regulatory or methodological context necessary to assess clinical translation. Priority was given to peer‐reviewed human studies reporting clinically interpretable outcomes. Purely conceptual reports, noncataract AI studies without direct translational relevance, duplicate reports without additional validation and studies reporting only training‐set performance were not prioritised.

The final synthesis included 24 sources, comprising cataract‐specific primary or technical studies, evidence reviews and meta‐analyses, epidemiologic context and regulatory/ethical guidance. Because the review was not prospectively designed as a systematic review, a formal PRISMA flow diagram and study‐level risk‐of‐bias instrument were not applied. To reduce subjective interpretation, however, evidence was appraised according to study design, dataset size and provenance, internal versus external validation, patient‐level versus image‐level evaluation where reported, comparator, performance metric, prospective evaluation and demonstrated clinical utility.

Clinical readiness was categorised as follows: Level 1, technical proof of concept or benchmark performance; Level 2, retrospective clinical development/internal validation; Level 3, external or multicentre validation; Level 4, prospective clinical evaluation or live clinical feasibility; and Level 5, routine clinical deployment with evidence of workflow or patient‐level performance. These levels are descriptive and are not intended as a validated grading system.

## 3. Results

### 3.1. AI in Cataract Detection and Diagnostic Imaging

Image‐based cataract detection is one of the most mature technical applications, but performance varies according to imaging modality, disease definition, reference standard and validation design. Shimizu et al. developed a DL approach for nuclear cataract assessment from slit‐lamp video [5]. Although the study demonstrated strong diagnostic performance, its principal contribution is technical validation; generalisability to different slit‐lamp systems, acquisition protocols and populations requires independent testing.

Tham et al. provide stronger evidence for generalisability. Their retinal photograph‐based DL algorithm was developed and tested using more than 25,000 images from population‐based cohorts and underwent external validation in three independent datasets [6]. The AUROC for visually significant cataract was 0.966 in the internal test set and 0.916–0.965 across external datasets, with sensitivity and specificity varying by cohort. This is more informative than a single accuracy value because it demonstrates both discrimination and performance variation across populations and grading systems. Importantly, external validation supports potential screening use but does not by itself establish that deployment improves referral appropriateness, visual outcomes, or cost‐effectiveness.

Ueno et al. evaluated a DL system for cataract and multiple anterior‐segment diseases using smartphone images [7]. In the smartphone test set, the reported AUROC for cataract was 0.992. This should not be interpreted as a ‘99% probability’ of correct diagnosis; AUROC describes discrimination across thresholds. Performance also depended on image quality and confidence thresholds, emphasising the need for acquisition standardisation and quality‐control mechanisms in real‐world use. Janti et al. subsequently reported approximately 90% overall accuracy in a prospective smartphone‐based cataract detection setting [8], supporting feasibility while still leaving questions about transportability, spectrum effects and downstream referral outcomes.

Taken together, cataract‐detection models have progressed from internal technical validation to external and prospective evaluation. However, claims that such systems can reliably replace ophthalmic examination are premature. Their most defensible current role is as screening or triage support in defined workflows with quality control and a clear referral pathway.

### 3.2. ML and Hybrid Approaches for IOL Power Calculation

IOL power calculation requires particularly careful terminology. Contemporary formulas include classical theoretical formulas, regression‐based methods, machine‐learning models, hybrid theoretical/data‐driven approaches and proprietary formulas whose internal architecture is incompletely disclosed. These categories should not be collapsed into a single ‘AI formula’ group.

Carmona‐González and Palomino‐Bautista developed the Karmona method using multiple ML models and reported 90% of eyes within ±0.50 D of target refraction in their study sample [9]. Zhou et al. reported favourable performance of AI‐derived formulas in highly myopic eyes in a systematic review and network meta‐analysis [10]. Li et al. evaluated the Nallasamy stacking ensemble in 6893 eyes and reported a mean absolute error of approximately 0.31 D, statistically outperforming several comparator formulas in that dataset [11]. However, the study used a single IOL model and lacked validation in an independent institution, limiting the strength of claims about broad superiority.

The Kane formula is frequently grouped with AI‐based methods because its developers describe the use of AI components, but its full architecture is proprietary and cannot be independently characterised in detail. It is therefore more precise to describe Kane as a proprietary contemporary formula that incorporates data‐driven/AI elements rather than as a transparent ML model.

Comparative IOL studies are highly sensitive to constant optimisation, axial‐length distribution, lens model, biometry device, prior corneal refractive surgery and statistical treatment of paired eyes. Reported differences between formulas are often small and may not generalise across devices or patient subgroups. Accordingly, the evidence supports incremental performance advantages for selected modern data‐driven formulas in defined cohorts rather than the broad claim that AI formulas consistently outperform conventional methods in all eyes.

### 3.3. Preoperative Risk Assessment and Patient‐Specific Planning

ML‐based perioperative risk models may support preoperative planning by combining multiple demographic, biometric, ocular and systemic variables. Gaskin et al. used a retrospective cataract‐surgery cohort to model individual complication risk [12]. Such models can identify associations and stratify risk, but predictive associations should not be interpreted as causal determinants or as evidence that an AI‐guided intervention improves outcomes.

Large registry datasets may improve statistical power but do not eliminate problems of dataset shift, missingness, coding quality, or local practice effects. Triepels et al. developed ML models using approximately 2.85 million EUREQUO cases to predict posterior capsule rupture [13]. This demonstrates the value of large‐scale registry modelling, but a registry‐derived probability estimate is not equivalent to real‐time intraoperative monitoring and should be presented as preoperative/perioperative risk stratification unless tested prospectively in live decision support.

Ghamsarian et al. analysed cataract surgery videos to quantify IOL unfolding delay, rotation and intraoperative instability and explored their relationship to postoperative lens stability [14]. The study supports automated characterisation of lens behaviour and hypothesis generation, but it does not justify stating that the model can yet predict late IOL dislocation for individual patients in routine practice. The appropriate interpretation is proof of concept for video‐derived intraoperative features that may be relevant to postoperative IOL stability.

At present, the strongest clinical use case for these models is risk stratification and decision support. Whether risk scores should alter surgeon assignment, equipment preparation, counselling or surgical technique requires prospective impact studies.

### 3.4. Intraoperative AI: Distinct Tasks and Evidence Levels

#### 3.4.1. Workflow and Phase Recognition

DeepPhase and related systems demonstrate that DL can recognise procedural phases from cataract‐surgery video [15]. A 2024 systematic review of AI‐based cataract video analysis found substantial methodological heterogeneity and highlighted reproducibility and reliability limitations across the literature [4]. Phase recognition can enable automated indexing, operative documentation and context‐aware systems, but recognition accuracy alone does not demonstrate improved surgical safety or patient outcomes.

#### 3.4.2. Anatomical and Instrument Segmentation

The CaDIS dataset and associated segmentation work established technical benchmarks for identifying instruments and ocular structures during cataract surgery [16]. These systems are enabling technologies for image‐guided surgery. Their clinical significance depends on whether segmentation remains robust under bleeding, corneal haze, pupil changes, instrument occlusion, variable microscopes and uncommon complications.

#### 3.4.3. Intraoperative Decision Support: Current Evidence and Limitations

Decision support should be distinguished from recognition. Within the prespecified search period, most cataract‐surgery AI evidence remained focused on offline workflow recognition, segmentation, registry‐based risk prediction, or other enabling technical tasks rather than live intraoperative intervention. A preoperative or registry‐derived probability estimate for posterior capsule rupture is not equivalent to real‐time risk monitoring, and technical recognition of instruments or anatomy does not itself demonstrate improved surgical safety. Real‐time proximity warnings, adaptive phaco settings and other context‐aware interventions should therefore be regarded as future‐facing applications unless tested prospectively in live surgery.

#### 3.4.4. Surgical Performance Assessment and Training

AI‐based analysis of cataract‐surgery video has also been explored for objective performance assessment and training, as summarised in prior reviews [3]. Potential metrics include workflow consistency, instrument handling and procedural timing. However, technical discrimination between levels of experience does not by itself establish educational benefit, credentialing validity or improved patient outcomes. Where automated ratings are compared with ophthalmologists or expert graders, the composition of the reference panel, reading conditions, rating instrument and statistical basis of agreement should be reported.

#### 3.4.5. Robotics and Force Feedback

Force‐feedback systems and steady‐hand robotics demonstrate that computer‐assisted microsurgery can improve precision in experimental settings [17, 18]. However, much of this evidence predates current cataract‐specific AI and derives from simulated or broader ophthalmic microsurgery. These technologies should therefore be presented as enabling or future‐facing platforms rather than established cataract‐care interventions.

### 3.5. AI‐Based Postoperative Monitoring and Follow‐Up

Postoperative AI includes image analysis, symptom triage and automated patient communication. Automated quantification of posterior capsule opacification has been explored using longitudinal slit‐lamp image analysis [19]. By contrast, routine autonomous detection of postoperative cystoid macular oedema on OCT specifically after cataract surgery remains insufficiently validated and should be regarded as a future application rather than an established clinical tool.

The strongest clinical evidence in this domain concerns conversational telephone follow‐up. Meinert et al. evaluated the autonomous assistant Dora in a multicentre cohort and reported 94% sensitivity and 86% specificity for identifying patients requiring further management [20]. These results support prospective clinical evaluation but do not establish universal replacement of conventional postoperative visits.

Accordingly, AI follow‐up should be described as a pathway component that may replace selected routine contacts in validated populations, not as a generic substitute for postoperative care. False‐negative consequences, emergency escalation, patient preference, accessibility, medicolegal responsibility and ongoing postdeployment auditing remain essential. The available evidence supports prospective clinical validation of selected telephone‐triage pathways, but not universal replacement of conventional follow‐up.

### 3.6. Evidence Summary and Clinical Readiness

Table 1 summarises representative evidence across the cataract pathway. The central pattern is a steep decline in evidence maturity from benchmark performance to demonstrated clinical benefit. External validation is increasingly available for diagnostic imaging, whereas IOL prediction remains highly dataset‐ and protocol‐dependent. Intraoperative computer vision is technically advanced but remains largely at the level of enabling technology within the prespecified search period. Conversational postoperative AI provides one of the clearest examples of progression to prospective multicentre clinical validation.

**TABLE 1:** Representative evidence across the cataract care pathway, classified by validation type and clinical‐readiness level (Level 1, technical proof of concept; Level 2, retrospective internal validation; Level 3, external/multicentre validation; Level 4, prospective clinical evaluation or live feasibility; Level 5, routine clinical deployment).

Application	Study, design and dataset	Validation	Key reported result	Principal limitation	Level	Interpretation
Cataract detection/grading	Shimizu et al. 2023 [5]; DL, slit‐lamp video, single‐centre development	Internal	Strong discrimination for nuclear cataract grading	Single acquisition setting; no independent testing	1‐2	Technical validation only
Cataract detection	Tham et al. 2022 [6]; DL, retinal photographs, > 25,000 images from population‐based cohorts	External (3 independent datasets)	AUROC 0.966 internal; 0.916–0.965 external	Referral, visual‐outcome and cost impact not assessed	3	Strongest generalisability evidence for detection
Cataract and anterior‐segment triage	Ueno 2024 et al. [7]; DL, smartphone and slit‐lamp images	Internal test set; multidisease	AUROC 0.992 for cataract (smartphone set)	Dependent on image quality and confidence thresholds	2‐3	Requires acquisition standardisation
Cataract detection (community setting)	Janti et al. 2024 [8]; prospective smartphone application evaluation	Prospective clinical	Approx. 90% overall accuracy	Accuracy is prevalence‐dependent; downstream referral not reported	4	Feasibility demonstrated; transportability unclear
IOL power calculation	Carmona‐González and Palomino‐Bautista 2021 [9]; ML‐based Karmona method	Internal	90% of eyes within ±0.50 D of target	Single‐centre; constant‐optimisation effects	2	Promising within development cohort
IOL power calculation (high myopia)	Zhou et al. 2023 [10]; systematic review and network meta‐analysis	Synthesis	Favourable ranking of AI‐derived formulas	Indirect comparisons; heterogeneous axial‐length distributions	n/a	Supports subgroup‐specific advantage only
IOL power calculation	Li and Nallasamy 2023 [11]; stacking‐ensemble Nallasamy formula, 6893 eyes	Internal	Mean absolute error ≈0.31 D vs comparator formulas	Single IOL model; no independent‐institution validation	2	Incremental, dataset‐specific advantage
Preoperative complication risk	Gaskin et al. 2016 [12]; retrospective predictive modelling of cataract‐surgery complications	Internal	Identification of risk factors and stratification	Associations only; no prospective impact evaluation	2	Risk stratification, not causal inference
Posterior capsule rupture risk	Triepels et al. 2023 [13]; ML on ≈2.85 million EUREQUO registry cases	Internal (registry)	Discrimination for posterior capsule rupture	Registry coding quality; not real‐time monitoring	2	Preoperative stratification only
Intraoperative IOL behaviour	Ghamsarian et al. 2024 [14]; DL video analysis of IOL unfolding, rotation, instability	Internal	Automated quantification of lens behaviour	Small event numbers; individual prediction unproven	1‐2	Hypothesis‐generating
Surgical phase recognition	Zisimopoulos et al. 2018 [15]; DeepPhase, cataract‐surgery video	Internal benchmark	Accurate recognition of procedural phases	No demonstrated safety or outcome benefit	1	Enabling technology
Instrument and anatomy segmentation	Grammatikopoulou et al. 2021 [16]; CaDIS benchmark dataset	Internal benchmark	Reference segmentation performance	Robustness under bleeding, haze, occlusion untested	1	Enabling technology
Intraoperative video AI (evidence synthesis)	Müller et al. 2024 [4]; systematic review	Synthesis	Substantial methodological heterogeneity	Limited reproducibility and reliability reporting	n/a	Cautions against pooled claims
Robotics and force feedback	Cutler et al. 2013 [17]; Taylor et al. 1999 [18]; simulated and experimental microsurgery	Experimental	Improved precision in simulation	Predates cataract‐specific AI; not cataract‐validated	1	Enabling platform
Posterior capsule opacification/anterior‐segment progression	Jiang et al. 2018 [19]; deep temporal sequence network on longitudinal slit‐lamp images	Internal	Prediction of anterior‐segment progression	Not independently validated in cataract‐specific deployment	1‐2	Developmental
Postoperative telephone triage	Meinert et al. 2024 [20]; autonomous assistant (Dora), multicentre cohort	Multicentre clinical	Sensitivity 94%, specificity 86%	Selected population; escalation dependent	4	Prospective validation achieved

## 4. Discussion

The literature supports a more cautious interpretation of AI in cataract care than a simple narrative of transition from prototype to routine use. Evidence maturity is heterogeneous. Large external validations exist for selected diagnostic models; data‐driven IOL formulas have shown small but meaningful improvements in defined datasets; intraoperative AI remains predominantly technical, with limited evidence beyond offline analysis or enabling computer‐vision tasks; and autonomous postoperative follow‐up has reached prospective multicentre validation in selected pathways. These achievements are substantial, but they do not justify treating all AI applications as clinically established.

Performance metrics must be interpreted in relation to study design. Accuracy can be misleading when disease prevalence or class balance differs between development and deployment populations. Sensitivity, specificity, AUROC, positive and negative predictive values, calibration and decision‐curve or clinical‐utility analyses provide complementary information. For predictive models, patient‐level splitting is generally preferable to image‐level splitting when multiple images from the same patient are present, because leakage can inflate apparent performance.

For IOL prediction, numerical superiority is particularly vulnerable to methodological choices. Constant optimisation, exclusion criteria, axial‐length distribution, IOL model, device‐specific biometry, prior refractive surgery and treatment of bilateral eyes can materially alter comparisons. A statistically significant difference in mean absolute error does not necessarily translate into a clinically important advantage for every population.

### 4.1. Dataset Quality, Bias and Generalisability

Common limitations across the evidence base include single‐centre retrospective development, incomplete demographic reporting, device‐specific image acquisition, selective exclusion of poor‐quality images, small or imbalanced complication cohorts, uncertain ground‐truth adjudication and limited external validation. Direct comparison with clinicians is also frequently absent or inadequately specified: where models are described as performing comparably to ophthalmologists, the number and experience of the comparator graders, the reading conditions, the information available to each arm and the statistical basis of the comparison should be reported. These factors can produce spectrum bias and reduce performance when models are transferred to different clinics, devices, ethnic groups, or disease prevalences.

Annotation reliability is especially important for cataract grading and surgical video analysis. Reference standards should describe grader expertise, adjudication of disagreements and intergrader variability. Imaging studies should report acquisition device, image‐quality criteria, preprocessing and whether multiple images from the same patient were separated between training and test partitions.

Dataset shift after deployment is an additional concern. Changes in camera hardware, IOL models, surgical technique, patient demographics, or clinical pathways may alter model performance over time. Clinical AI therefore requires ongoing monitoring rather than a one‐time validation.

### 4.2. Regulation, Human Oversight and Postmarket Monitoring

AI systems used for diagnosis, surgical planning, or postoperative triage may meet the definition of medical device software depending on intended use. Regulatory assessment therefore extends beyond algorithm accuracy to clinical evaluation, risk management, usability, cybersecurity, change management and postmarket surveillance.

In the European Union, medical‐device AI may be subject to existing medical‐device legislation and, where applicable, the AI Act, which establishes harmonised requirements for high‐risk AI systems, including risk management, data governance, technical documentation, transparency and human oversight [21]. This is particularly relevant for systems that influence diagnosis or treatment decisions and illustrates why regulatory classification depends on intended use rather than on the use of AI alone.

Regulatory considerations also differ between static models and systems that may be modified after deployment. Under the EU Medical Device Regulation, software qualifying as a medical device is subject to classification according to intended purpose, conformity assessment, clinical evaluation and postmarket surveillance obligations, and substantial modifications to a certified device may require renewed conformity assessment [22]. Material model updates, new input modalities or deployment in materially different populations may therefore alter performance and require renewed validation, governance review and, where applicable, regulatory reassessment. Postmarket surveillance is particularly important because dataset shift can occur even when the underlying algorithm is unchanged.

Human oversight should be explicit. Clinical interfaces should define when an AI output is advisory, when it triggers escalation, who is responsible for reviewing uncertain or discordant cases, and how model failures are documented. Explainability methods may assist reviewers in interrogating individual outputs, but they do not substitute for external validation or prospective evaluation [23]. Ophthalmology‐specific ethical analyses emphasise transparency, informed consent, equity of access, automation bias and unresolved moral and legal responsibility when an autonomous or semiautonomous system contributes to a clinical decision [24]. Liability is likely to depend on intended use, regulatory status, local governance and whether clinicians use the system consistently with validated indications.

### 4.3. Clinical Integration and Utility

An AI model can be technically excellent yet clinically unhelpful if it adds workflow friction, duplicates existing information or generates alerts without a clear action pathway. Studies should therefore evaluate time saved, changes in referral or complication‐detection rates, clinician workload, patient acceptability, false‐negative consequences and cost‐effectiveness in addition to discrimination metrics.

Postoperative conversational AI illustrates this distinction. The clinically meaningful question is not whether a language model can classify symptoms in isolation, but whether the complete pathway (automated contact, escalation, clinician review, urgent access and audit) maintains or improves safety while reducing unnecessary clinician‐led encounters.

### 4.4. Future Directions: Explicitly Unvalidated or Emerging Applications

Several frequently discussed applications remain plausible but insufficiently validated for routine use. These include preoperative prediction of phacoemulsification energy or ultrasound time; automated selection of chopping strategy or phaco settings; personalised recommendation of multifocal, extended‐depth‐of‐focus or toric IOLs based on lifestyle and predicted satisfaction; real‐time proximity warnings for the posterior capsule or iris; autonomous prediction of postoperative dissatisfaction; and automated detection of cataract‐specific postoperative macular complications from OCT.

These concepts should be tested in staged translational studies. A reasonable sequence is technical development, independent external validation, prospective silent evaluation, interventional clinical testing and postdeployment monitoring. Studies should prespecify clinically meaningful endpoints rather than infer patient benefit from technical accuracy alone.

Some of these concepts may progress rapidly as surgical video datasets and real‐time computing mature. Nevertheless, future claims should remain proportional to the level of validation available within the relevant evidence base and should not infer patient benefit from technical feasibility alone.

### 4.5. Limitations of This Review

This review remains narrative and therefore does not provide the exhaustive study identification, duplicate screening, formal risk‐of‐bias assessment or meta‐analytic pooling expected of a systematic review. Study identification and screening were performed by a single author, with uncertain cases resolved by discussion among the coauthors; the absence of duplicate independent screening means that selection bias cannot be excluded. The search was limited to English‐language literature and focused on clinically relevant cataract applications. The proposed readiness hierarchy is a pragmatic descriptive framework rather than a validated grading tool. Rapid publication in this field also means that regulatory status and the level of deployment of specific products can change after the search date.

## 5. Conclusion

AI in cataract care spans a broad hierarchy of evidence. Externally validated image‐based cataract detection is technically mature, but real‐world screening benefit remains context dependent. Data‐driven and hybrid IOL formulas can improve refractive prediction in selected cohorts, yet comparisons require rigorous control of optimisation and biometric confounders. Intraoperative AI remains primarily at the level of phase recognition, segmentation, risk modelling and other enabling technical tasks, with limited evidence of reduced complications or improved patient outcomes. Autonomous postoperative follow‐up has reached multicentre prospective validation in selected pathways, but this does not establish universal replacement of conventional follow‐up.

The major translational gaps are therefore not simply algorithm development, but independent external validation, representative datasets, prospective clinical utility, workflow integration, transparent human oversight, regulatory compliance and postmarket monitoring. Clinical adoption should be driven by demonstrated benefit in the intended population and pathway rather than by benchmark performance alone.

## Author Contributions

Writing of the original draft, A.J.; review and editing, B.Đ.I. and R.K.Ž.; supervision, J.P. and T.J.

## Funding

The authors received no specific funding for this work.

## Disclosure

All authors have read and agreed to the published version of the manuscript.

## Conflicts of Interest

The authors declare no conflicts of interest.

## Supporting Information

Additional supporting information can be found online in the Supporting Information section.

## Supporting information


**Supporting Information** Appendix S1. Structured Search Strategy.

## Data Availability

Data sharing is not applicable to this article, as no new data were created or analysed. All studies discussed are published and are listed in the reference list, and the full search strings used to identify them are provided in the Supporting Information.
