# Supplementary material for: Artificial Intelligence in Cataract Care: A Narrative Review of Evidence Maturity and Clinical Readiness
Source: J Ophthalmol. 2026 Nov 1;2026:8100180. doi: 10.1155/joph/8100180 (PMC13725069; doi:10.1155/joph/8100180)
Supplement: Supplementary file 1 [file JOPH-2026-8100180-s001.docx]

## Supporting Information: Appendix S1. Structured Search Strategy

**Search 1. General cataract AI:** ("cataract"[Title/Abstract] OR "cataract surgery"[Title/Abstract]) AND ("artificial intelligence"[Title/Abstract] OR "machine learning"[Title/Abstract] OR "deep learning"[Title/Abstract] OR "neural network"[Title/Abstract])

**Search 2. IOL prediction:** ("cataract surgery"[Title/Abstract] OR "intraocular lens"[Title/Abstract] OR IOL[Title/Abstract]) AND ("machine learning"[Title/Abstract] OR "artificial intelligence"[Title/Abstract] OR "deep learning"[Title/Abstract]) AND (calculation[Title/Abstract] OR prediction[Title/Abstract] OR refraction[Title/Abstract])

**Search 3. Intraoperative AI:** ("cataract surgery"[Title/Abstract]) AND ("artificial intelligence"[Title/Abstract] OR "machine learning"[Title/Abstract] OR "deep learning"[Title/Abstract]) AND ("surgical video"[Title/Abstract] OR "phase recognition"[Title/Abstract] OR segmentation[Title/Abstract] OR intraoperative[Title/Abstract] OR robot*[Title/Abstract])

**Search 4. Postoperative monitoring:** ("cataract surgery"[Title/Abstract]) AND ("artificial intelligence"[Title/Abstract] OR "machine learning"[Title/Abstract] OR "natural language processing"[Title/Abstract]) AND (postoperative[Title/Abstract] OR follow-up[Title/Abstract] OR telemedicine[Title/Abstract] OR triage[Title/Abstract])

Filters/prioritization: English language; publication date 1 January 2018–31 March 2025. Earlier seminal studies were retained selectively when directly relevant to cataract risk prediction, force-feedback systems, or ophthalmic robotics.
